# Engineered Cell Membrane-Derived Nanocarriers: The Enhanced Delivery System for Therapeutic Applications

**DOI:** 10.3389/fcell.2022.844050

**Published:** 2022-02-28

**Authors:** Biao Yu, Xu Xue, Zhifeng Yin, Liehu Cao, Mengmeng Li, Jianping Huang

**Affiliations:** ^1^ The Second Affiliated Hospital, Shanghai University, Shanghai, China; ^2^ School of Medicine, Shanghai University, Shanghai, China; ^3^ Institute of Translational Medicine, Shanghai University, Shanghai, China; ^4^ Department of Orthopedics, Shanghai Zhongye Hospital, Shanghai, China; ^5^ Department of Orthopedics, Luodian Hospital, Shanghai, China; ^6^ Department of Orthopedics, Luodian Hospital, Shanghai University, Shanghai, China; ^7^ Department of Neurology, Wenzhou Central Hospital, Wenzhou, China

**Keywords:** multi-functionality, membrane engineering, drug delivery, targeting effect, cell membrane camouflaged nanoparticles

## Abstract

There has been a rapid development of biomimetic platforms using cell membranes as nanocarriers to camouflage nanoparticles for enhancing bio-interfacial capabilities. Various sources of cell membranes have been explored for natural functions such as circulation and targeting effect. Biomedical applications of cell membranes-based delivery systems are expanding from cancer to multiple diseases. However, the natural properties of cell membranes are still far from achieving desired functions and effects as a nanocarrier platform for various diseases. To obtain multi-functionality and multitasking in complex biological systems, various functionalized modifications of cell membranes are being developed based on physical, chemical, and biological methods. Notably, many research opportunities have been initiated at the interface of multi-technologies and cell membranes, opening a promising frontier in therapeutic applications. Herein, the current exploration of natural cell membrane functionality, the design principles for engineered cell membrane-based delivery systems, and the disease applications are reviewed, with a special focus on the emerging strategies in engineering approaches.

## 1 Introduction

Alongside the rapid advances in nanotechnology, nanoparticles (NPs) have attracted a great deal of attention in medical research and showed remarkable advantages in terms of efficacy and safety in comparison to existing therapeutic and diagnostic methods (Li et al., 2015; Ragelle et al., 2017; Fang et al., 2018). An effective biological interface is a prerequisite for the successful transformation of experimental materials *in vivo* (Cai et al., 2018; Metavarayuth et al., 2019). Once the nanoparticles enter the body, they are exposed to a complex environment that could recognize and eliminate foreign elements (Yoo et al., 2011; Zou et al., 2021). Therefore, researchers have designed nanoparticles with the ultimate goal of making their surfaces ignorable by all objects except the target, and achieving this goal has proven to be extremely difficult.

As the most basic unit of life, the cells grow with a multitude of complex physiological activities, and perform various functions by interaction and exchange with surroundings (Chen et al., 2018a; Chen et al., 2020a). Moreover, the cell membrane, located at the outermost layer, takes the primary responsibility (Li X. Q. et al., 2020). The phospholipid bilayers, proteins and carbohydrates are major components of the cell membranes (Hu et al., 2021a; Chen et al., 2022; Gao et al., 2022). The main function of lipids is to maintain the bilayer structure and fluidity of the cell membrane (Pomorski et al., 2001). Proteins and carbohydrates are essential for the interfacial interaction, particularly for signal recognition. Moreover, the cell membrane carries many natural “self markers” such as CD47, CD44 proteins and glycans, which enable the nanoparticles to escape from immunogenic clearance (Oldenborg et al., 2000; Dahl et al., 2002). Notably, most cancer cells display homologous targeting ability due to the presence of specific membrane proteins such as N-cadherin, galectin-3, and epithelial cell adhesion molecules (EpCAM) (Fang et al., 2014).

Faced with the challenge of functionalization strategies for synthetic nanoparticles, researchers try to combine artificially synthesized nanoparticles with natural biomaterial coatings to develop a new bionic delivery platform (Yoo et al., 2011; Dehaini et al., 2016). The advantage of this strategy is the customizability and versatility of synthetic materials, as well as the functionality and complexity of natural biomaterials. Natural cell membrane-coated nanoparticles are of particular interest due to their potential to create new therapies. The cell membrane-encapsulated nanoparticles essentially inherit the biological characteristics of the parent cell membranes, such as self-labeling and homologous targeting (Hu et al., 2013; Piao et al., 2014; Kroll et al., 2017). To obtain diverse functions, hybrid membrane-encapsulated nanoparticles can be developed by fusing multiple cell membranes, which inherit the delicate affinity ligand inherent in the parental cell (Chen et al., 2020b).

Notably, the convergence of multiple modification techniques and cell membranes has provided tremendous promise for cell membrane-encapsulated delivery systems. Physical, chemical and biological engineering approaches can be adopted to obtain multifunction and improve the targeting effect of the cell membrane. For example, lipid insertion is directly applied to modify cell membranes (Zhang M. et al., 2021; Ai et al., 2021). Moreover, the cell membrane can be genetically engineered to express specific markers for targeting therapeutics. Remarkable advances in this field have stimulated the interest of many researchers to expand the range of cell membrane bionic delivery systems through physical, chemical and biological engineering strategies.

In this paper, we will introduce functionalization related to prolonging systemic circulation and cell-specific targeting of natural and engineered cell membrane-encapsulated nanoparticles. Significantly, we emphasized the design principle of establishing additional functions of cell membrane-encapsulated nanoparticles and discussed the advantages and limitations of the engineering methods and their biomedical application. Furthermore, we summarized underlying mechanisms for emerging advances in cell membrane-encapsulated nanoparticles and discussed the physical, chemical and biological engineering approaches in the design of functionalization for cell membrane encapsulated nanoparticles.

## 2 Preparation of Cell Membrane-Coated Nanoparticles

### 2.1 Acquisition of Cell Membrane Coatings

The cytoplasmic membrane is a phospholipid bilayer structure with various proteins and carbohydrates which are essential in cell growth and development, especially in cell recognition. Therefore, it is extremely important to maintain the integrity of the cell membrane structure at the moment of isolation and purification of cell membranes. To obtain the complete cell membrane structure, cells may be subjected to repeated freezing and resuscitation, hypotonic treatment, mechanical extrusion, and in the case of nucleated cells, removal of their complex contents (Guo et al., 2018; Oieni et al., 2020). Large quantities of parent cells can be obtained in cell culture or directly from a tissue sample, and then after completing the above steps, pure cell membranes can be obtained by gradient centrifugation to remove material other than cell membranes (Pomorski et al., 2001). Newly prepared cell membranes should be used immediately or stored at 80°C to maintain their biological activity, sometimes with the addition of protease inhibitors to prevent the degradation of membrane proteins (Gao et al., 2015).

### 2.2 Method of Coating

Extrusion, ultrasound and electroporation methods are commonly used to prepare cell membrane-encapsulated nanoparticles (Chen et al., 2017). Briefly, the extrusion method includes mixing cell membranes and nanoparticles, and then squeezing the mixture through polycarbonate membranes repeatedly at least five times with different pore sizes to form particles of the desired size (Rao et al., 2020). The sonication method utilizes electrostatic interactions, and the mixture of cell membranes and nanoparticles is prepared by certain power and intermittent ultrasound to wrap the cell membranes around the nanoparticles (Wei et al., 2016). This method exhibit simplicity in operation, but tends to make the membrane unevenly distributed (Vijayan et al., 2018). In the process of electroporation, several small pores can be created in the cell membrane for a transient period under an external electric field (Wei et al., 2018). These pores allow the entrance of nanoparticle cores or drug molecules as well as the outflow of intracellular material, thus leading to production of cell membrane-encapsulated nanoparticles. However, this method causes minimal damage to the cell membrane itself. Recently, researchers constructed thin-layer evaporation methods and microfluidic-based methods for the preparation of biomimetic nanovesicles (Zinger et al., 2021a; Zinger et al., 2021b). Notably, the biomimetic nanovesicles using microfluidic approach present reproducible and uniform in size, and the microfluidic method can be used for large-scale production without impairing the function of cell membrane proteins (Molinaro et al., 2018).

## 3 Functions of Nanoparticles Encapsulated With Natural Cell Membrane

### 3.1 Monotypic Cell Membrane-Encapsulated Nanoparticles

#### 3.1.1 Prolonging Systemic Circulation

Among the available coating materials, polyethylene glycol (PEG) is most commonly used for extending the blood circulation of nanoparticles (Knop et al., 2010). Polyethylene glycol could produce a hydrated layer and provide spatial stabilization, thus impeding the interaction between the environment. PEG has been successfully used in a variety of clinical products (Hu et al., 2014). However, it was found that the first injection of polyethylene glycol-modified nanoparticles could lead to immune responses that induce the production of anti-polyethylene glycol immunoglobulin M antibodies (Zhang et al., 2016). Therefore, researchers are trying to explore natural membranes for stealth coatings.

Erythrocytes, natural long-circulation transporters, are most abundant in blood and can be retained in the human body for up to 120 days (Muzykantov, 2010). In addition, mature erythrocytes have no nucleus or complex organelles, making it easy to obtain a pure cell membrane (Wibroe et al., 2017). Moreover, the erythrocyte membrane itself has a multitude of natural “self markers” such as CD47 proteins, glycans and acidic silyl molecules which enable its encapsulated nanoparticles to evade immunogenic clearance and provide extending circulation times for the nanoparticles (Oldenborg et al., 2000; Dahl et al., 2002; Ke et al., 2022). Therefore, the erythrocyte membrane is an ideal material for nanoparticle surface modification.

**FIGURE 1:**
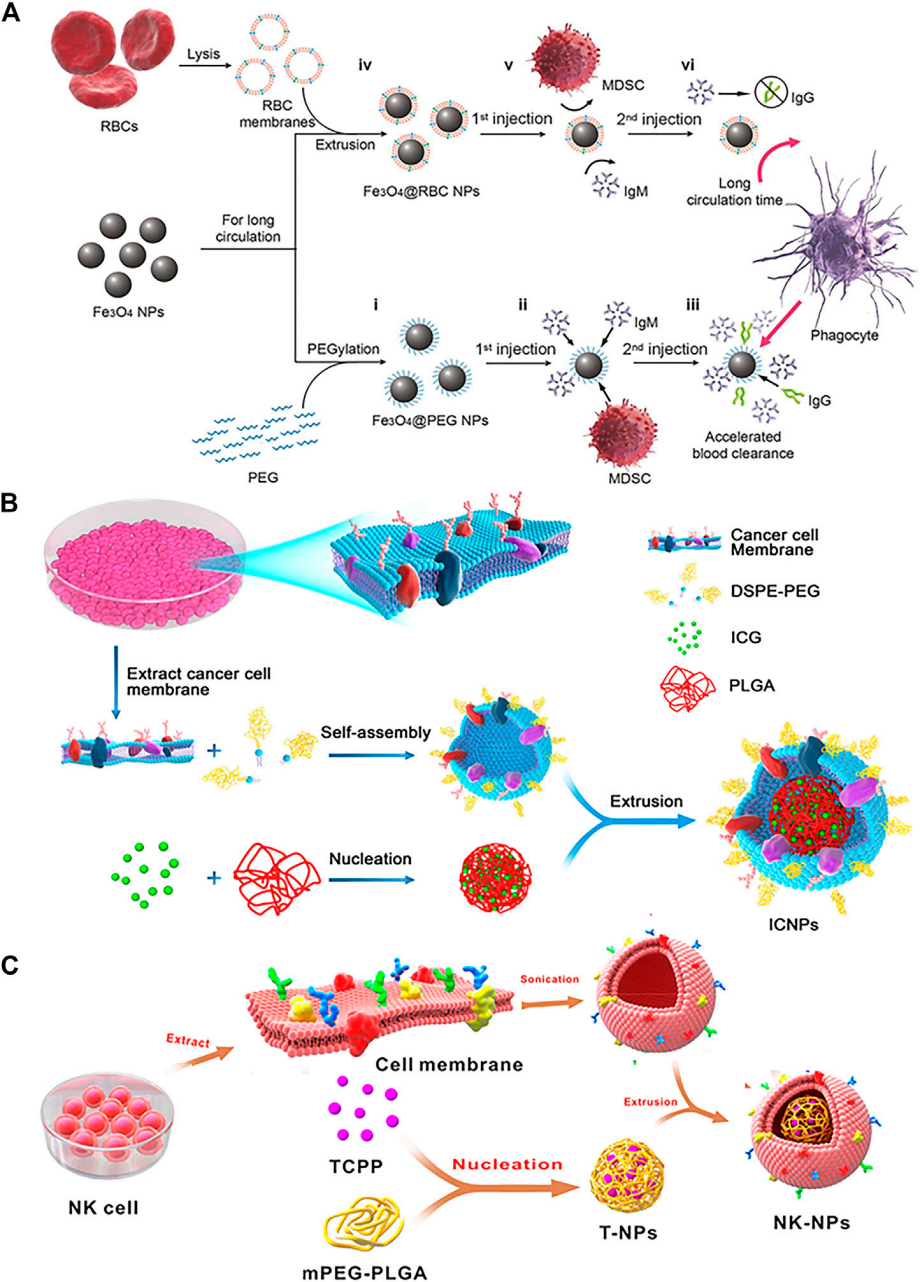
**(A)** Schematic of preparations of Fe_3_O_4_@PEG and Fe_3_O_4_@RBC NPs and the events after they are injected into the blood (Reproduced with permission from Rao et al. (2015)). **(B)** The schematic preparation procedure of ICNPs. Fusing cancer MCF-7 cell membrane and PEGylated phospholipids (DSPE-PEG) and then coating onto ICG-loaded polymeric cores by extrusion. (Reproduced with permission from Chen et al. (2016)). **(C)** Schematic Illustration of NK Cell-Membranes-Cloaked Nanoparticles(NK-NPs) (Reproduced with permission from Deng G. et al. (2018)).

Erythrocyte membrane-encapsulated nanoparticles are the first system of mimic cell membranes, which is reported in 2011 by the team of Liangfang Zhang, and are currently the most common natural carrier for biomedical applications (Hu et al., 2011). Rao et al. (2015) demonstrated that the natural biofilm of red blood cells (RBCs) was more conducive to immune escape compared to PEG through a comparative test of bionic RBC membranes encapsulated with Fe_3_O_4_ nanoparticles and PEG-modified Fe_3_O_4_ nanoparticles (Figure 1A). Macrophage uptake and pharmacokinetic studies demonstrated the superiority of RBC membranes over PEG in prolonging cycle times. The Fe_3_O_4_ (Fe_3_O_4_@ RBC) NPs could escape immune clearance by relying on the CD47/SIRP-α signaling approach. The Fe_3_O_4_@RBC NPs obtained a longer cycle and no“accelerated blood clearance (ABC)” occurred. Furthermore, Fe_3_O_4_@RBC NPs did not induce immune response at the cellular level (myeloid-derived suppressor cells (MDSCs)) or the humoral level (immunoglobulins M and G (IgM and IgG)). Furthermore, the results of blood biochemical, hematological and histological assays showed no significant toxicity of the erythrocyte membrane-encapsulated nanoparticles *in vivo*.

Cheng et al. constructed the bio-nanoparticles (QD@P)Rs by encapsulating Pluronic F-127-modified Ag2S QDs in erythrocyte vesicles for enzyme-augmented non-invasive sonodynamic therapy (SDT) (Li et al., 2020). The *in vitro* and *in vivo* experiments indicated that (QD@P)Rs had favorable biocompatibility and could distinctly prolong circulation. Kui et al. utilized two different blood cell membrane-encapsulated mesoporous silica nanoparticles to deliver nanoparticles with fibrinolytic activity [fullerenol (Fols)] for the treatment of blood clots (Chen et al., 2020c). The results showed that erythrocyte membrane-encapsulated nanoparticles exhibited longer blood circulation time compared to platelet membrane-encapsulated nanoparticles. In fact, natural cell membranes-camouflaged nanoparticles have the ability to evade immune clearance and prolong blood circulation, however, the ability of red blood cells in immune evasion is most prominent. In other words, erythrocyte membranes can be used instead of PEG to help nanoparticles escape immune clearance to prolong blood circulation.

### 3.1.2 Cell-Specific Targeting

Some types of cell membranes were used to encapsulate nanoparticles due to the ability to target without additional modification. Inherent cell adhesion characteristics play an important role in such targeting, particularly in cancer. In tumors, numerous cancer cells display homotypic targeting through surface-specific antigens with homophilic adhesion domains (Fang et al., 2014). Therefore, the use of cancer cell membrane-coated nanoparticles can perfectly replicate various surface antigens from cancer cell membranes to gain the ability to escape immune clearance and homologous targeting thus being considered as promising cancer therapies (Oldenborg et al., 2000; Dahl et al., 2002).

To explore the natural ability of cancer cell interactions, nanoparticles ICNPs were obtained by extruding cancer MCF-7 cell membranes onto indocyanine green (ICG)-loaded PLGA nanoparticles (Figure 1B) (Chen et al., 2016). The experimental results showed that ICNPs significantly targeted and accumulated at the tumor site due to the inheritance of homologous binding adhesion molecules on the membrane surface of cancer cells *in vivo*. In addition, ICNPs are adept at masquerading as cells to reduce liver and kidney interception. In another study, Fang et al. (2014) demonstrated the homologous targeting of cancer cell membrane-encapsulated nanoparticles (CCNPs) derived from breast cancer cell membranes encapsulated with PLGA nuclei. The results revealed the CCNPs had higher binding and uptake capacity compared to bare PLGA nuclei and RBC-NPs in live MDA-MB-435 cells. Notably, this connection was cell-specific as there was no increased binding of CCNPs to heterotypic human foreskin fibroblasts compared to naked PLGA cores.

Bone marrow-derived natural killer (NK) cells, the core cells of the natural immune system, are the body’s first line of defense against cancer cells and viral infections (Smyth et al., 2002). In tumor immunotherapy, NK cells not only induce M1-type polarization of macrophages to secrete proinflammatory cytokines and chemokines that participate in the positive immune response and function as immune surveillance but also target tumor sites through proteins on the NK cell membrane (Fang et al., 2016; Wan et al., 2016; Huang et al., 2017). Notably, NK cells recognition of self and non-self is dependent on the expression of multiple receptors (Ljunggren and Kärre, 1990). Therefore, NK cell membrane-encapsulated nanoparticles can induce M1 polarization of macrophages and target tumor cells. Besides, it can also act as a membrane inducer to stimulate the immune system. In one case, the researchers successfully prepared NK cell membrane-encapsulated photosensitizer 4,4′,4′′,4′′′-(porphine5,10,15,20-tetryl) tetrakis (benzoic acid) (TCPP)-loaded nanoparticles (NK-NPs) to improve the efficacy of NK cell-membrane immunotherapy (Figure 1C) (Deng G. et al., 2018). The results showed that NK-NPs were able to specifically accumulate at tumor sites. Moreover, NK-NPs-mediated PDT could boost NK cell membrane immunotherapy which could eradicate the primary tumor and inhibit the growth of distant untreated tumors.

In addition to erythrocyte and tumor cell membranes, several other cell membranes have been used for nanoparticle platforms such as platelet membranes, macrophage membranes, leukocyte membranes, natural killer cell membranes, T cell membranes, monocyte membranes and dendritic cell membranes. Recently, novel delivery platforms based on exosomes and bacterial extracellular vesicles are extraordinarily hot, which also broaden the selectivity of biofilms (Song et al., 2019a; Hu et al., 2021b; Liu et al., 2021; Pan et al., 2021). This is a summary of cell membrane-derived wrapped core particles for the treatment of various in Table 1.

**TABLE 1:** Summary of the membrane sources, core particles and effect of different cell membrane camouflaged nanoparticles.

Source of cell membranes	Core particle	Effect	Disease	Ref.
RBCs	PLGA	Absorbing membrane damaging toxins	Toxin-mediated	Ben-Akiva et al. (2020)
	PLGA	Targeting tumor	Human lung cancer (A549)	Chai et al. (2017)
	Hydroxycamptothecin	Extending circulation time	Human cervical cancer (HeLa)	Ye et al. (2019)
		Improving tumor accumulation		
	Ag2S quantum dot	Extending circulation time	Mouse colon cancer (C26)	Li et al. (2020)
		Biocompatibility		
	Gold nanowire motor	Absorbing membrane damaging toxins	Toxin-mediated	Wu et al. (2015)
	Oncolytic adenovirus	Targeting tumor	Human liver cancer (HepG)	Lv et al. (2019)
	Dimeric prodrug	Extending circulation time	Human cervical cancer (HeLa)	Pei et al. (2018)
		Improving tumor accumulation		
	Prussian blue	Evading immune clearance	Human breast cancer (4T1)	Chen et al. (2017)
	Oil nanodroplet	Absorbing membrane damaging toxins	Toxin-mediated	Chen et al. (2019)
	Melanin	Extending circulation time	Human lung cancer (A549)	Jiang et al. (2017)
		Improving tumor accumulation		
	Iron oxide	Evading immune clearance	Human breast cancer (MCF-7)	Ren et al. (2016)
	Magnetic mesoporous silica	Evading immune clearance	Human breast cancer (4T1)	Xuan et al. (2018)
		Improving tumor accumulation		
Cancer cells	Polyamidoa-mine dendrimer	Targeting tumor	Human lung cancer (H1975)	Wu et al. (2019)
	PLGA	Targeting tumor	Human liver cancer (HepG2)	Liu et al. (2019d)
	PLGA	Targeting tumor	Human breast cancer (MDA-MB-231)	Jin et al. (2019)
	Gold nanocage	Targeting tumor	Mouse breast cancer (4T1)	Sun et al. (2020)
	Bovine serum albumin-drug nanocrystal	Targeting tumor	Mouse breast cancer (4T1)	Zhang et al. (2019)
	Copper sulfide	Targeting tumor	Mouse melanoma (B16-F10)	Wu et al. (2020a)
	Porphyrin-based metal organic framework	Targeting tumor	Mouse breast cancer (4T1)	Li et al. (2017)
	Gelatin	Targeting tumor	Patient-derived squamous carcinoma	Rao et al. (2019)
	Lipoplex	Targeting tumor	Breast cancer (4T1, MDA-MB-831)	Kroll et al. (2017), Kumar et al. (2019b), Kim et al. (2019)
	Rare-earth doped nanoparticles	Targeting tumor	Human breast cancer (MDA-MB-231)	Zhang et al. (2020)
	Poly(epsilon-caprolactone)	Targeting tumor	Human glioblastoma (U87)	Wang et al. (2020)
Platelets	PLGA	Evading immune clearance	Mouse liver cancer (H22)	Wang et al. (2019)
		Targeting tumor		
	PLGA	Evading immune clearance Subendothelium binding	Coronary restenosis	Chen et al. (2020c)
		Pathogen adhesion		
	Magnetic nanoparticles	Homing to atherosclerotic sites	Atherosclerosis	Song et al. (2019b)
	Magnetic nanoparticles	Specific clearance of anti-platelet antibodies	Immune thrombocytopenia purpura	Wei et al. (2016)
	Magnetic nanoparticles	Evading immune clearance	Mouse breast cancer (4T1)	Jiang et al. (2020)
		Targeting tumor		
	Polypyrrole	Evading immune clearance	Human liver cancer (Huh 7)	Wu et al. (2020b)
		Targeting tumor		
	Mesoporous silica	Extending circulation time Target accumulation	Carotid thrombosis	Chen et al. (2020c)
Macrophages	Silica NPs	Cytocompatibility	Rheumatoid arthritis	Fontana et al. (2018)
	Au nanoshells	Targeting tumor	Mouse breast cancer (4T1)	Xuan et al. (2016)
Leukocytes	Silica NPs	Cancer cell targeting	Human cervical cancer (HeLa)	He et al. (2016)
	(Alginate/chitosan) 8 capsules	Evading immune clearance	Inflammation	Gao et al. (2016)
		Improving tumor accumulation		
Natural killer cells	Liposome	Targeting tumor	Human breast cancer (MCF-7)	Pitchaimani et al. (2018)
	PLGA	Targeting tumor	Human breast cancer (4T1)	Deng J. et al. (2018)
T cells	PLGA	Targeting tumor	Human lymphoma (Raji)	Han et al. (2019)
	PLGA	Decoys for viral attack and neutralize	HIV infection	Wei et al. (2018)
		HIV		
Monocytes	PLGA	Targeting tumor	Human breast cancer (MCF-7)	Krishnamurthy et al. (2016)
Dendritic cells	Metalorganic framework	T cell activation	Mouse breast cancer (4T1)	Liu et al. (2019b)

### 3.2 Hybrid Cell Membrane-Encapsulated Nanoparticles

The achievement of a single type of cell membrane-encapsulated nanoparticles has boosted the progress of fusing multiple cell membranes for functionalization of nanoparticles (Dehaini et al., 2017). Compared with monotypic cell membranes, the hybridized cell membranes endow the synthesized nanoparticles with more biological functions obtained from the original cells. Taking advantage of the functional complementarity, hybrid membrane-encapsulated nanoparticles inherit the characteristics of each parental cell type (Dehaini et al., 2017). The hybrid membrane-encapsulated nanoparticles have better performance compared to the same monotypic cell membrane type in therapeutic applications (He et al., 2018; Rao et al., 2018; Bu et al., 2019; Jiang et al., 2019; Liu et al., 2019a; Ye et al., 2020).

**FIGURE 2:**
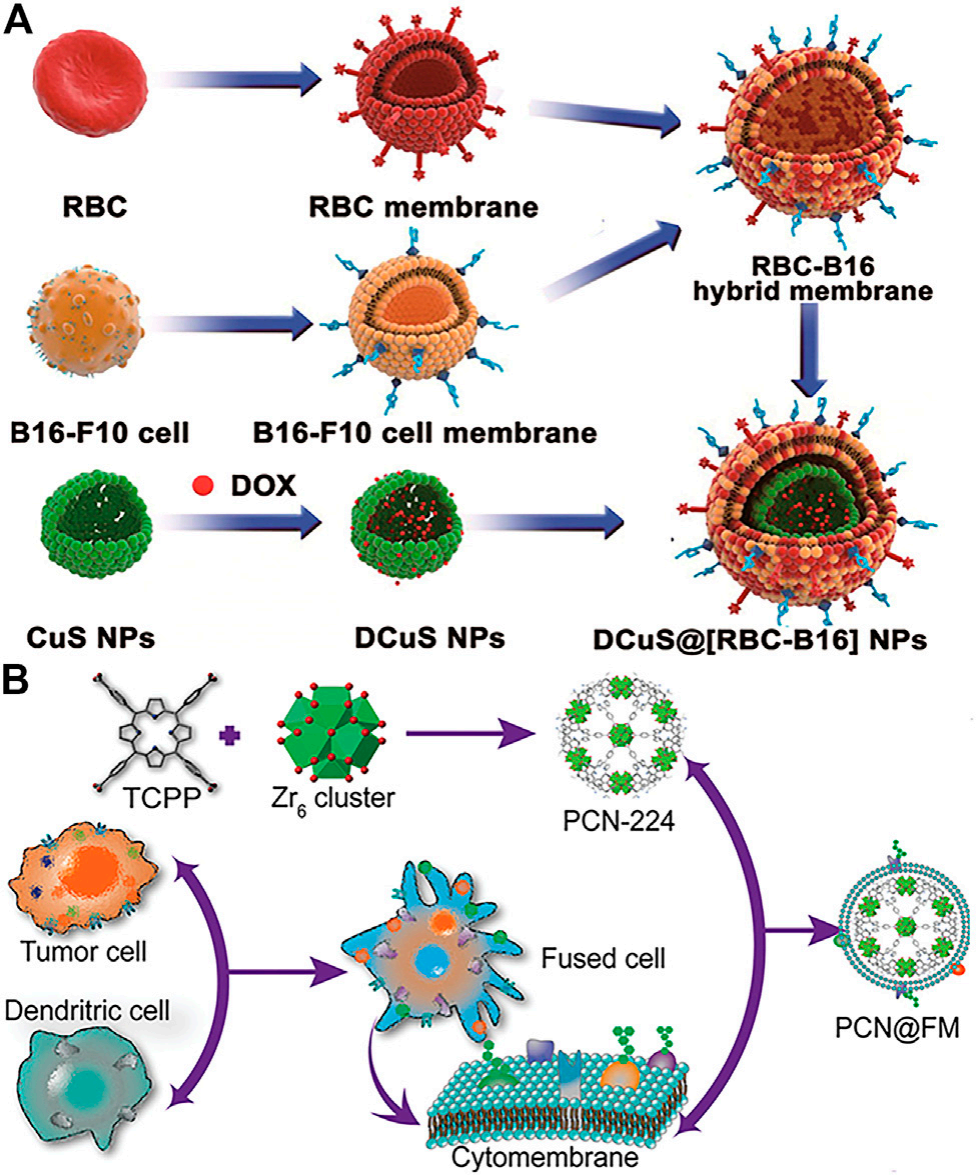
**(A)** Schematic of preparations of DCuS@[RBC-B16] NPs. The fusion membranes of RBCs and B16F10 cells are encapsulated on DOX-loaded hollow copper sulfide nanoparticles (DCuS NPs) (Reproduced with permission from Wang et al., (2018a)). **(B)** Illustration of preparation process of PCN@FM for monotherapy (Reproduced with permission from Liu et al. (2019a)).

Nanoparticles encapsulated by cancer cell membranes alone have displayed significantly homotypic targeting to source tumors. However, the efficacy of nanoparticles encapsulated only by cancer cell membranes is limited by the lack of sufficient stealth to evade immune clearance mainly due to the presence of tumor-specific antigens on the membrane surface (Shi et al., 2017; He et al., 2018). To solve this problem, a method was developed to coat nanoparticles using fused membranes of erythrocytes and cancer cells, which combines the functions of both cell membranes (Wang et al., 2018a; Jiang et al., 2019; Xiong et al., 2021). For example, RBC and B16-F10 cancer cell membranes were mixed to create RBC-B16 hybrid biofilm-coated hollow copper sulfide nanoparticles (DCuS@[RBC-B16] NPs) loaded with doxorubicin for combination photothermal/chemotherapy of melanoma (Figure 2A) (Wang et al., 2018a). The results showed that DCuS@[RBC-B16]NPs inherited the characteristic functions of both parent cells. The incorporation of erythrocyte membranes carried a large number of “self-tagged” proteins, significantly enhancing the immune evasion ability of DCuS with longer blood circulation times, while the B16-F10 cancer cell membrane coating enhanced the homogeneous targeting ability of melanoma due to the presence of homologous adhesion protein molecules.

Membrane hybridization can improve the immune escape of nanoparticles by introducing another membrane with greater stealth capabilities. Moreover, it can also fuse two or more different cell type-specific affinity ligands to provide hybrid membrane-encapsulated nanoparticles with multiple targeting capabilities. Wen et al. wrapped porphyrin-based ZrMOF (PCN224) with hybrid membranes of tumor cells and dendritic cells for tumor treatment (Liu et al., 2019a). The hybrid membrane inherited the homologous antigens of tumor cells as well as the specific tumor recognition antigens of dendritic cells. The results showed that the hybrid membrane-induced immunotherapy showed superb anti-tumor effects, even comparable to PDT. In addition, membrane hybridization can be used to introduce specific antigens and increase immunostimulatory properties to improve immunotherapy (Liu et al., 2019b). Bacterial membranes have also been used for hybrid functionalization, stimulating immune maturation and preventing tumorigenesis (Chen et al., 2020d). Except for hybridization among natural membranes, liposomes are also favorable by researchers as the functionalized groups on liposomes could fuse into cell membranes by simple preparation (Lv et al., 2020; Zhang M et al., 2021).

There is a summary of different types of hybridized membrane-functionalized nanoparticles in Table 2. Researchers have investigated multiple membrane combinations that produce synergistic effects by combining different functions (Dehaini et al., 2017; Wang et al., 2018a; Liu et al., 2019a). Due to the diversity of cell membranes, membrane hybridization will provide tremendous flexibility for designing individualized nanodrugs. It is believed that membrane hybridization technology will have promising applications in the pharmaceutical field.

**TABLE 2:** Summary of different types of hybridized membrane-functionalized nanoparticles.

Hybrid membrane sources	Core particle	Effects	Disease model	Ref.
Erythrocyte-platelet cells	PLGA	Prolonged circulation time	Human breast cancer (MDA-MB-231)	Dehaini et al. (2017)
		Enhanced tumor accumulation		
Erythrocyte-cancer cells	Melanin nanoparticles	Prolonged circulation time	Mouse melanoma (B16-F10, MCF-7), ovarian cancer (ID8)	Wang et al. (2018a), Jiang et al. (2019), Xiong et al. (2021)
		Enhanced tumor accumulation		
	Fe_3_O_4_ magnetic nanoparticles			
Leukocyte-platelet cells	Immunomagnetic beads	Prolonged circulation time	Breast cancer	Rao et al. (2018)
		Improved isolation of circulating tumor cells		
Leukocyte -cancer cells	Paclitaxel (PTX)	Prolonged circulation time	Head and neck cancer (HN12)	He et al. (2018)
		Enhanced tumor accumulation		
Platelet-cancer stem cells	Fe_3_O_4_ magnetic nanoparticles	Prolonged circulation time	Head and neck squamous cell carcinoma (CAL27)	Bu et al. (2019)
		Improved isolation of circulating tumor cells		
Platelet-neutrophil cells	Gold nanocage	Prolonged circulation time	Human breast cancer (MDA-MB-231) Mouse breast cancer (4T1)	Ye et al. (2020)
		Improved isolation of circulating tumor cells		
Cancer-dendritic cells	Porphyrin-based Zr-MOF (PCN-224)	Prolonged circulation time	Mouse breast cancer (4T1)	Liu et al. (2019a)
		Enhanced tumor accumulation		
Bacteria- cancer cells	PLGA-ICG (PI)	Stimulated immune maturation	Mouse melanoma (B16-F10)	Chen et al. (2020d)
Liposome- cancer cells	lipoic acid-modified polypeptide (LC) micellar system	Enhanced tumor accumulation	Human non-small cell lung cancer (A549)	Zhang M. et al. (2021)
		Multiple modified liposomes bring various functions together		
Exosome–liposomes	Granulocyte-macrophage colony-stimulating factor (GM-CSF)	Enhanced tumor accumulation	Metastatic peritoneal cancer (CT26)	Lv et al. (2020)
	Docetaxel (DTX)			

## 4 Engineered Cell Membrane-Encapsulated Nanoparticles

Recently, membrane engineering has been used to directly or indirectly modify natural cell membranes for obtaining multifunctionalized membrane-encapsulated nanoparticles. In brief, the direct modification of cell membranes focuses on the integration of specific ligands onto the cell membrane coating to target specific receptors of the aimed cell by physical or chemical methods (Zhou et al., 2016; Zhang M. et al., 2021). Indirect modifications mainly involve manipulating the natural biosynthetic pathways or modifying the genes of cells at the molecular or cellular level to make cell membranes functionalized through metabolic engineering techniques and genetic engineering techniques.

### 4.1 Physical Modification

Physical modification of cell membranes encapsulated with nanoparticles is a non-covalent modification that is mild and harmless compared to chemical modifications and can preserve the activity of cell membrane surface proteins (Zhang M. et al., 2021). The most commonly used physical modification method is lipid insertion. The functional fragments could be spontaneously attached or inserted into phospholipid bilayers via a hydrophobic bond (Marqués-Gallego and de Kroon, 2014). Sonication and extrusion are the most commonly used methods for lipid insertion, by which the ligand density on the membrane can be modulated for formulation optimization by controlling initial input (Wang et al., 2015). The ligands with different molecular weights are also applicable for the modification of the membrane. These merits altogether have led to the widespread interest in functionalizing cell membrane-encapsulated nanoparticles by lipid insertion.

**FIGURE 3:**
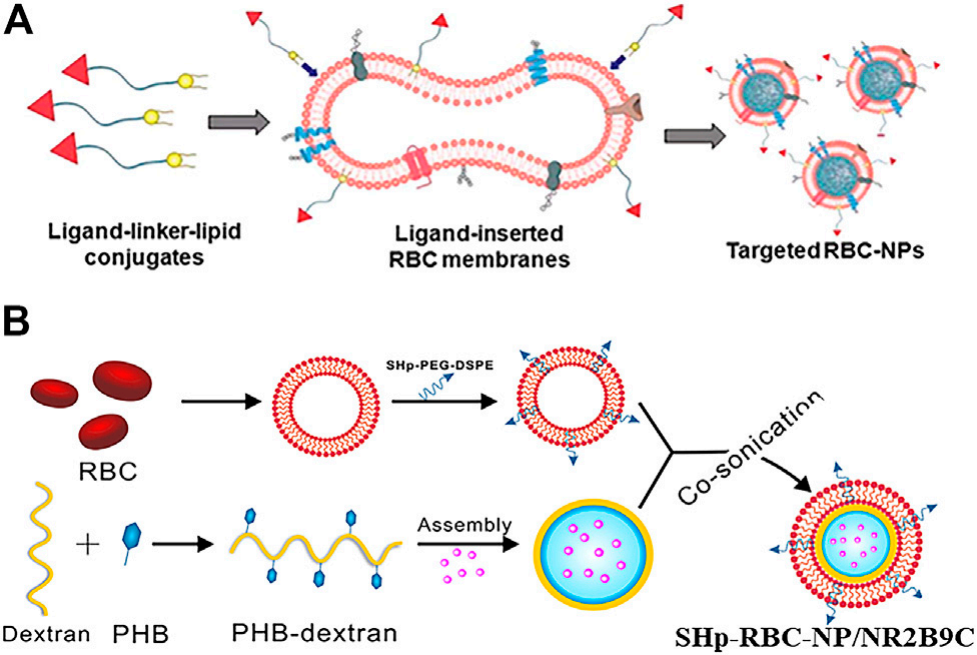
**(A)** Schematic of the preparation of targeted RBC-NPs (Reproduced with permission from Fang et al. (2013)). **(B)** Schematic of the preparation of SHp-RBC-NP/NR2B9C (Reproduced with permission from Lv et al. (2018)).

Incorporating the anchor ligands into the lipid for targeting has been commonly used because of its simplicity in operation and its controllability of conjugation effects. For example, Fang et al. (2013) studied the fluidity of bilayered RBC membranes and developed an approach to protecting existing surface proteins. As shown in Figure 3A, targeting moieties were integrated onto bilayered membranes with the assistance of lipid tethers. The ligand–linker–lipid conjugates were inserted into the RBC membranes, which functionalized RBC membranes by protecting the membrane from chemical reactions. Moreover, the researchers compared the receptor-specific targeting ability of functionalized RBC-NPs with two differently sized ligands (MW ∼ 441 Da; MW ∼ 9,000 Da) in model cancer cell lines, demonstrating that the technique can be applied to ligands of different weight scales. The 1,2-distearoyl-sn-glycerol-3-phosphoethanolamine-N-[amino(polyethylene glycol)-2000] (PEG-DSPE) is mostly used as the lipid anchor, with a PEG spacer added to preserve the freedom of the ligand for bioactivity (Guo et al., 2015; Mac et al., 2016; Rao et al., 2017; Ak et al., 2018; Deng G. et al., 2018; Yang et al., 2018; Zhu et al., 2018; Fu et al., 2019; Kumar et al., 2019a; Guliz and Sanlier, 2020). Targeted delivery strategies have been applied to more types of diseases such as melanoma, glioblastoma, and stroke (Liu et al., 2018; Lv et al., 2018; Wang et al., 2018b; Zou et al., 2018; Liu et al., 2020). For example, Lv et al. (2018) developed a ROS-responsive nanocarrier loaded with a neuroprotective agent (NR2B9C) that could target the site of the stroke to treat ischemic brain injury. Stroke homing peptides (SHp) were conjugated to PEG-DSPE and then inserted into the RBC membrane to encapsulate the dextran polymer core modified with ROS-responsive boronic ester (Figure 3B). The SHp-RBC-NP/NR2B9C obtained the targeting ability to the ischemic brain site by SHp mediated transcytosis and extended the circulation life via the RBC membrane. After being phagocytosed into ischemic neurons, the high levels of intracellular ROS could promote the release of the neuroprotective agent NR2B9C from SHp-RBC-NP/NR2B9C to exert its therapeutic effect.

In addition to ligands, antibodies can also be anchored to the surface of cell membranes for targeting by lipid insertion. The prerequisite is that the lipid molecule is first attached to functional groups that could recognize antibodies including aldehydes, amines, thiols, carboxyl groups, and thiols (Li et al., 2018; Zhang M. et al., 2021). Moreover, the lipid insertion can alter the properties of the cell membrane after inserting by carrying functions that are responsive to stimulation such as oxygen levels, light, and pH in the microenvironment (Su et al., 2016; Liu et al., 2019c; Dong et al., 2020). Therefore, this lipid insertion modified cell membrane-encapsulated nanoparticles can also be used to facilitate a two-step “pre-targeting” strategy to enrich and image the tumor microenvironment (Li M. et al., 2020).

Overall, as a non-disruptive functionalization strategy, lipid insertion has successfully integrated various affinity ligands into the cell membrane to obtain the desired targeting ability. The summary of the introduction of different types of ligands into cell membrane coatings for functionalization by lipid insertion was demonstrated in Table 3. Hydrophobic bonds cause less damage to the inherent physicochemical properties of surface proteins on cell membranes compared to chemical conjugation. However, the inserted targets usually lack stability. Different from the large transmembrane part of proteins, inserted ligands usually have a hydrophobic domain similar to lipid alkyl chains, thus trending to leave cell membranes in the case that plenty of proteins appears nearby (Li et al., 2018).

**TABLE 3:** Summary of the introduction of different types of ligands into cell membrane coatings for functionalization by lipid insertion.

Membrane source	Ligand	Spacer	Target cell (receptor)	References
RBCs	AS1411 aptamer	PEG2000	Breast cancer cell (nucleolin)	Fang et al. (2013)
	Folate	PEG2000	Breast cancer cell cervical cancer cell ovarian cancer cell (folate receptor)	Rao et al. (2017), Ak et al. (2018), Deng J. et al. (2018), Kumar et al. (2019a), Guliz and Sanlier (2020)
	Mannose	PEG2000	Antigen-presenting cell (mannose receptor)	Guo et al. (2015)
	cRGD	PEG2000	Melanoma cell (αvβ3 integrin)	Wang et al. (2018b), Liu et al. (2018)
	Angiopeptide 2	PEG2000	Glioblastoma cell (LRP receptor)	Zou et al. (2018), Liu et al. (2020)
	T7/NGR peptide	PEG2000	brain endothelial cell (transferrin receptor) glioblastoma cell (CD13)	Fu et al. (2019)
	Stroke homing peptide	PEG2000	Apoptotic neuron cell (glutamate receptor)	Lv et al. (2018)
	Anti-HER2	PEG2000	Ovarian cancer cell (HER2)	Mac et al. (2016)
	Biotinylated anti-EpCAM	PEG2000-biotin-avidin	Breast cancer cell (EpCAM)	Zhu et al. (2018)
	Anti-EGFR-iRGD	PEG3400	Gastric cancer cell (EGFR, αvβ3 integrin)	Chen et al. (2018b)
	Biotinylated c(RGDyK)	PEG3400-streptavidin	Tumor vasculature endothelial cell, glioma cell (αvβ3 integrin)	Chai et al. (2019)
Cancer cells	Mannose	PEG2000	Dendritic cell (mannose receptor)	Yang et al. (2018)
	Anti-TGFβRII	PEG2000-azobenzene	Hypoxia-triggered release of TGFβ-neutralizing antibody	Dong et al. (2020)

### 4.2 Chemical Modification

Chemical modifications modify the cell membrane surface of cell membrane-wrapped nanoparticles mainly through covalent bonds, which provide a more stable anchoring (Zhou et al., 2016). A huge number of molecules in the cell membrane offer a wide variety of modification sites. The proteins and polysaccharides on the cell membrane can be targets sites due to the functional groups in response to various chemical reactions, such as primary amines, carboxylic acids, and thiols, providing covalent bonds for the desired adducts (Li et al., 2018). Notably, primary amines are the most commonly used functional groups among these functional groups.

#### 4.2.1 Primary Amine-Carboxylate Reaction

Previous studies have found the presence of a large number of activated primary amine groups which involve the formation of amide bonds by reacting with activated carboxylic acid groups in the outer leaflet of the cell membrane (Smarr et al., 2011). Patel et al. (2019) introduced maleimide (Mal) to the surface of bacterial membrane-coated nanoparticles (BNPs) to enhance antigen uptake through a reaction between Mal-PEG4-NHS and the amine groups on the bacterial membrane protein. In this chemical reaction, the carboxyl group should be converted to a chloride, which is an unstable intermediate susceptible to hydrolysis. The chloride hydrolysis influences production efficiency. Therefore, NHS was introduced to modify the molecule to convert the carboxylic acid group into a relatively stable group to improve the stability of the reaction.

#### 4.2.2 Biotin-Avidin Binding

Biotin-avidin binding is also a commonly used method for modifying cell membranes. In this method, Chai et al. (2017) used the strong binding affinity between biotin and avidin so that ^D^CDX peptides can attach to biotin molecules and bind to the surface of RBC membrane-coated nanoparticles (RBCNPs). Biotin was first anchored to the cell membrane to construct biotinylated cell membrane-coated nanoparticles (CM-NPs). Then, the biotinylated moiety was bound to streptavidin and anchored to the modified molecule. The avidin can bind biotin molecules multivalently, thus improving the active targeting efficiency of ^D^CDX - RBCNPs. Strongly positively charged targeting ligands are less susceptible to lipid insertion because of possible electrostatic interactions with negatively charged groups on cell membranes. The introduction of avidin, a larger protein molecule, into the cell membrane can serve as a means to shield the electrostatic binding between the positive charge peptide and the negative charge cell membrane. However, because of the immunogenic nature of affin/streptavidin, biotin-affinity binding activates immune clearance and is not a suitable method for application in clinical therapy (Chinol et al., 1998).

#### 4.2.3 Thiol-Maleimide Reaction

The cell membranes of T cells, hematopoietic stem cells, and B cells have a large number of thiol groups, which can be combined with sulfhydryl-reactive maleimide groups in the form of covalent bonds, so that various synthetic maleimide groups can be attached to the cell membranes (Stephan et al., 2010). In this approach, Cheng et al. (2012) completed the first step of cell membrane modification of cell membrane-coated nanoparticles (CM-NPs) using the NHS-PEG2-maleimide, and then further treated the cells with peptide ligands, thus successfully coupling the peptide ligands to the cell membranes.

#### 4.2.4 Click Chemistry Reaction

Zhang et al. (2017) successfully modified antigen-presenting cells (APCs) with therapeutic molecules by click chemistry reaction. Firstly, the azide (N_3_) was integrated into the leukocyte membrane through natural biosynthesis and metabolic incorporation of phospholipids. The engineered leukocyte membrane fragments were then used to wrap magnetic nanoclusters with superparamagnetic and magnetically responsive properties. Subsequently, N3-labeled magnetic nanoclusters coupled with major histocompatibility complex class I (pMHC-I) and co-stimulatory ligand anti-CD28 via click chemistry. The nanoclusters could promote the proliferation of CD8^+^ T cells in the presence of both ligands as compared to free anti-CD28. When the T cells were intravenously injected *in vivo*, the mice treated with T cells activated by nanoclusters demonstrated slower tumor growth as well as a better survival rate when compared with that activated by free antibodies.

However, some membrane proteins may be inactivated and lose their functions due to the lack of specificity of covalent modifications which makes it possible for the reactive groups to react with the proteins on the cell membrane. Therefore, it is necessary to improve the existing chemical modification methods or develop a better one.

### 4.3 Biological Modification

#### 4.3.1 Metabolic Engineering

Metabolic engineering achieves modification of the cell membrane by manipulating the natural biosynthetic pathways of the cell (Ai et al., 2021). The functional part is first bound to the metabolic substrate and then taken up into the cell to participate in the metabolic pathway during cell incubation (Agatemor et al., 2019; García-Granados et al., 2019). The functional part is not completely degraded or lost during metabolism but is anchored to the cell membrane when the metabolic substrate is involved in cell membrane formation (Du et al., 2009; García-Granados et al., 2019). Both glycoengineering and lipid engineering are based on this principle for membrane modification.

Glycoengineering can utilize the production of oligosaccharides and glycoconjugates to accomplish modifications of cell membranes such as the fucose salvage pathway. Monosaccharide substrates are typically used to form a binding to a metabolic functional part such as N-acetylmannosamine (ManNAc), GalNAc, and fucose (Cheng et al., 2016; Zhang et al., 2017; Han et al., 2019). Similarly, lipid engineering utilizes the synthetic pathways of natural lipids to achieve membrane functionalization, portions of which are typically bound to choline analogs for metabolism. Multiple functional moieties were mounted on the membrane surface to obtain the desired function through metabolic engineering, specifically bioorthogonal linkers (Nilsson et al., 2020).

**FIGURE 4:**
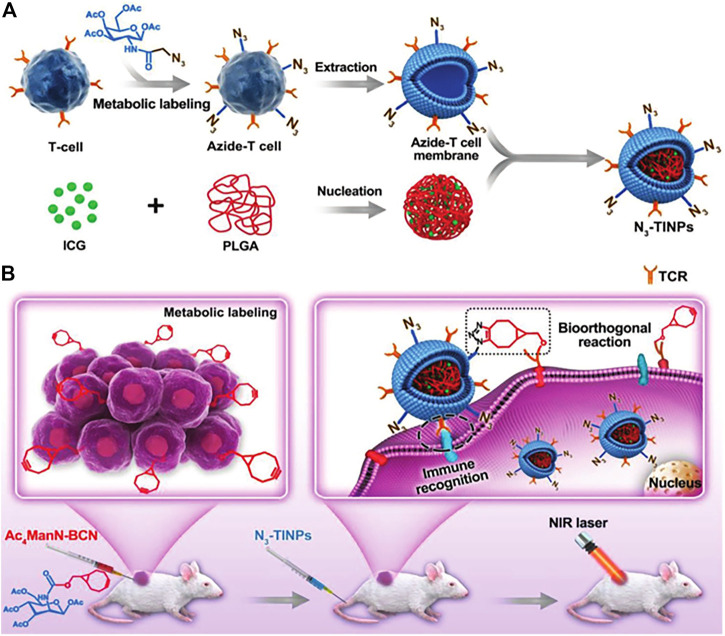
Schematic of N_3_-labeled T cell-encapsulated nanoparticles (N_3_-TINPs) with a dual-targeting mechanism for highly efficient photothermal therapy. **(A)** Preparation of N_3_-TINPs. **(B)** N_3_-TINPs could target tumors for phototherapy through immune recognition of T cell membranes and bio-orthogonal responses of BCN and N_3_ groups. (Reproduced with permission from Han et al. (2019)).

In metabolic engineering, glycoengineering is often applied to obtain the ability to target tumors. Non-natural glucose modified with azide (N_3_) or bicyclo[6.1.0]nonane (BCN) has been successfully inserted into the surface of various tumor cells to provide additional targeting capabilities through natural glycophysiological processes (Li et al., 2019). Han et al. (2019) successfully constructed N_3_-labeled T cell membrane-encapsulated ICG-PLGA nanoparticles (N_3_-TINPs) using the natural glycophysiological pathway which can specifically target natural antigens and BCN artificial receptors on tumors through immune recognition and bioorthogonal chemistry (Figure 4). The experimental results indicated that the fluorescence intensity in tumors of mice treated with N_3_-TINPs was 1.5 times higher than the mice treated with unlabeled TINPs. Moreover, the selective accumulation of N3-TINPs at tumor sites greatly improved the photothermal efficacy and effectively reduced the adverse side effects.

Similarly, phospholipid engineering techniques could introduce bio-orthogonal linkers on membrane-encapsulated nanoparticles to obtain targeting ability. Zhang et al. (2017) pre-engineered leukocyte membranes with azide (N_3_) through inherent biosynthesis and metabolic binding of phospholipids, dibenzocyclooctane-modified T-cell stimulators can be modified by copper-free click chemistry. Subsequently, N3-labeled magnetic nanoclusters coupled with major histocompatibility complex class I (pMHC-I) and co-stimulatory ligand anti-CD28 via click chemistry induced a significant increase of CD8^+^ T cell proliferation compared to free anti-CD28. The results showed that this nanocluster was effective in delaying tumor growth with fewer side effects in the murine EG7-based model.

Moreover, metabolic engineering makes clever use of natural biosynthetic pathways to anchor the ligands on cell membranes for camouflaging nanoparticles. With the discovery of novel ligands compatible with biosynthesis and the development of methods to enhance ligand expression, functional applications in metabolic engineering are expected to develop rapidly. When enough natural synthetic pathways are available, different ligands can be used in different ways to achieve multifunctional modifications of cell membranes, allowing for a wider range of applications of nanoparticle platforms.

#### 4.3.2 Genetic Modification

Gene modification is the expression of the desired product on the surface of the cell membrane via transcription and translation by selective gene editing. The modified cell membrane is then encapsulated in nanoparticles for functionalization. The modified T cells can target tumor-associated antigens by the introduction of artificial T cell receptor genes known as chimeric antigen receptors (CARs) (Jiang et al., 2016). Following retroviral transduction, CAR-T cells capable of stably expressing antigen receptors can be used to provide bionic membranes to camouflage nanoparticle cores (Ma et al., 2020). Common gene-editing methods include viral transfection and physical methods. In recent years, CRISPR/Cas9 technology has developed rapidly, making gene editing not only simpler and more efficient but also cheaper and more accurate (Deng et al., 2015; Meaker et al., 2020).

##### 4.3.2.1 Prolonging Systemic Circulation

**FIGURE 5:**
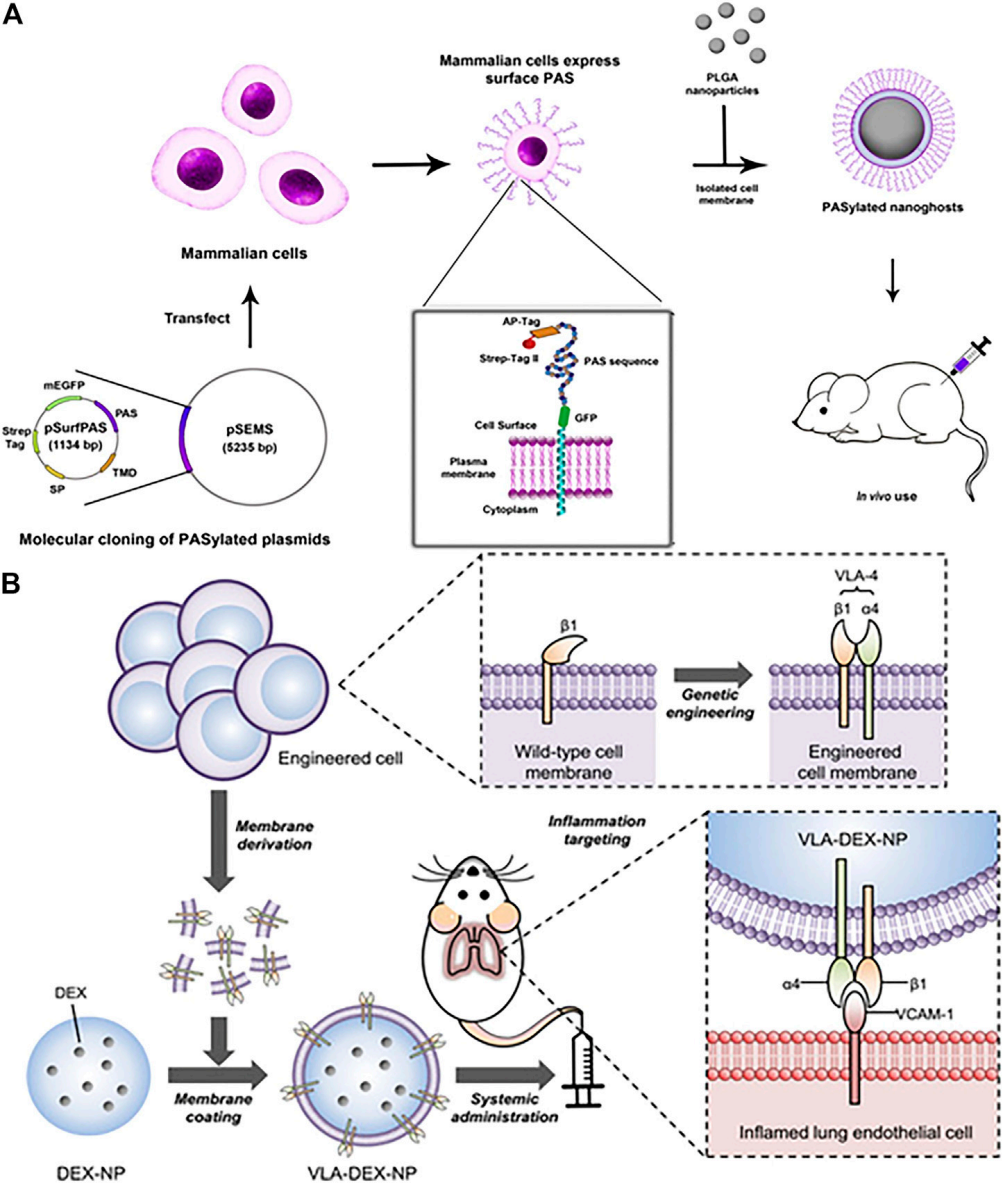
**(A)** Illustration of functionalized PASylated nanoghosts obtained by plasmid transfection. (Reproduced with permission from Krishnamurthy et al. (2019)). **(B)** Schematic illustration of genetically engineered cell membrane–coated nanoparticles for targeted DEX to inflamed lungs. (Reproduced with permission from Park et al. (2021)).

Functionalized cell membrane-encapsulated nanoparticles by genetic modification can significantly prolong the circulation time compared to wild-type cell membrane-encapsulated nanoparticles. Traditionally, polyethylene glycol is the gold standard that has been used to prolong the somatic circulation of nanoparticles. However, the applications of PEG have been limited due to issues such as immunogenicity, which is prompting researchers to look for alternative strategies (Garay et al., 2012). Recently, PASylation has been reported to be used to extend the circulation time of protein biologics *in vivo* (Schlapschy et al., 2013). PASylation refers to the addition of a conformationally disordered polypeptide chain to the N-terminal or C-terminal end of protein therapeutics, which includes repeating sequences of proline, alanine, and serine (PAS). Notably, it has superior biophysical properties compared to PEG. Krishnamurthy et al. (2019) successfully used plasmid transfection to express PAS chains on HEK293 cells and then wrapped the modified cell membranes around PLGA cores for functionalization (Figure 5A). The results showed that membrane expression of PAS 40 repeats reduced protein binding, reduced macrophage uptake by 90% compared to non-PASylated controls, and prolonged circulating half-life, resulting in an approximately seven times increase serum concentrations at 24 and 48 h *in vivo*. The overexpressed PAS on cell membranes is most likely a choice for polyethylene glycolization for camouflaging nanoparticles to evade immune clearance by genetic engineering.

##### 4.3.2.2 Enhanced Cell-Specific Targeting

The scheme is also feasible to express ligands with high affinity on the cell membrane surface by genetic modification to obtain targeting ability. This is a common feature that vascular cell adhesion molecule-1 (VCAM-1) expression is obviously upregulated when endothelial cells undergo inflammation (Kelly et al., 2007; Kourtzelis et al., 2017). For example, the specific affinity between very late antigen-4 (VLA-4) and VCAM-1 was used to develop nanoparticles for targeted treatment of inflammation(Figure 5B) (Park et al., 2021). The VLA-4 is composed of integrins α4 and β1 together, whereas wild-type cells express only β1. Wild-type cells were successfully expressed VLA-4 by genetic modification, and their cell membranes were subsequently coated on dexamethasone-loaded nanoparticle cores (DEX-NP) for anti-inflammatory purposes. The results showed that cell membrane-encapsulated DEX-NP (VLA-DEX-NP) with high expression of VLA-4 could significantly target inflammatory lung endothelial cells through VLA-4 and VCAM-1 specific affinity while enhancing drug delivery. In another study, Lv et al. (2019) used genetic engineering to express hepatitis B virus (HBV) preS1 ligands on HepG2 cells, and then encapsulate the membranes on lysing adenovirus, exhibiting liposome-like nanostructures and efficient tumor targeting specificity.

Gene modification holds great promise for creating nanoparticles with “universal” membranes due to the use of allogeneic cells after selective knockdown of antigen-presenting proteins such as MHC I and II. However, gene modification is a more complicated process compared with other membrane modification methods, and it is difficult to ensure the stable expression of some goal genes. Therefore, there are not many applications of genetic engineering yet.

## 5 Challenges for Clinical Translation

Existing studies have explored various laboratory-viable methods for functionalization of natural membranes, however, it is still difficult to produce cell membrane-encapsulated nanoparticles with homogeneous and stable physicochemical properties on a large scale. The research on the biomedical applications of cell membrane-encapsulated nanoparticles is still at the laboratory stage. For instance, red blood cell membrane-encapsulated nanoparticles were used to treat autoimmune hemolytic anemia and overcome the apparent toxic side effects of clinical hormone shock therapy (Copp et al., 2014). And studies related to the application areas of certain types of cell membranes are still insufficient such as mesenchymal stem cell membranes used for treatment of various diseases (Xu and Li, 2014). Moreover, a comprehensive study of the biological properties of membrane-encapsulated nanoparticles is needed, especially related to the safety of cancer cell membrane-derived drug delivery systems. In addition, cell membranes are inadequately sourced, and the timeliness and efficacy of preparation should be considered when using patients’ cells, so a reliable set of conditions and evaluation criteria for clinical formulation parameters is important. Therefore, the preparation process should be optimized and parameters during membrane functionalization should be determined for facilitating clinical translation in future studies. Due to the lack of basic research on cell membranes, immunogenicity is a great challenge when using cell preparations from different sources. Although there are many studies on natural membrane-encapsulated nanoparticles, there is no successful clinical translation yet. Encouragingly, with the help of lipid nanocarriers, three siRNA drugs and two new coronavirus mRNA vaccines have now been approved and are available worldwide. In addition, the clinical trial application of SQZ Biotech’s erythrocyte vector tumor vaccine SQZ-AAC-HPV has been approved by the U.S. FDA and is currently in the first phase of clinical studies. It is believed that functionalized membranes will make a big impact on clinical practice in the future such as drug-carrying coatings (Wang and Tang, 2019; Zhang W et al., 2021).

## 6 Conclusion and Perspectives

Owing to the inherent biological characteristics and excellent biocompatibility of natural cell membranes, as well as the customizability and flexibility of nanoparticles, functionalized cell membrane-camouflaged nanoparticles have been widely studied for medicine delivery, imaging, detoxification, detection, and enabling photosensitized therapies. However, the lack of functionality of natural cell membranes has limited their widespread use within complex physiological environments. To address this issue, researchers have performed direct and indirect modifications to cell membranes such as lipid insertion and gene editing. Compared to direct modification of isolated cell membranes, modification of living cell membranes before extraction has several advantages including that 1) separation of the modified living cell from the free unbound molecules is simpler, preserving the function of membrane structure well and saving time, 2) molecular anchoring is more stable and interactions between anchored molecules strengthen the formation of functionalized cell membranes, 3) modification of living cell membranes ensures the correct orientation for anchoring, whereas partially directly modified cell membranes exhibit an inside-out orientation, which may result in the opposite orientation of the functional domains and thus dysfunction. Nanoparticles coated with cell membranes can retain the physicochemical characteristics of the nanoparticle core, as well as inherit the biological functions of the parent cells. Recently, with the increasing cellular membrane sources and functionalization methods, the combination of physical, biological, and chemical methods is being developed to achieve the desired multi-functionality of cell membranes. Additionally, membrane coatings are used not only for higher dimensional biomaterials but also for autonomous propulsion of nanomotors. The functionalized modification and application of membrane coatings will become more widespread when a breakthrough in basic membrane research can effectively address the immunogenicity of allogeneic cells. Moreover, the functionalization of nanoparticles cannot be limited to membrane coating alone. The modification of the nucleus and membrane can synergistically promote the multi-functionalization of membrane-coated nanocarriers. Optimizing the composition of membrane source and nanoparticle cores can also meet the needs of different properties. For example, the loading can be adjusted by adjusting the surface charge, size, structure or hydrophobicity of the core material. Furthermore, the current disease applications are not limited to tumors, but also include various diseases such as inflammation and thrombosis. In the future, functionalized cell membrane-coated nanoparticles have great promise in blood-related diseases due to the natural advantages of cell membranes. It is believed that the rapid and high-quality development of medical, nanotechnology, material science, bioengineering and pharmaceutical disciplines can greatly facilitate the application of engineered cell membrane-derived nanocarriers.
